# Unilateral Hypoglossal Nerve Palsy in a Patient with a Difficult Airway Requiring Prolonged Intubation

**DOI:** 10.1155/2021/8842503

**Published:** 2021-02-18

**Authors:** Kahlin Leuzinger, Lopa Misra

**Affiliations:** Mayo Clinic Hospital, 5777 E. Mayo Blvd., Phoenix, AZ 85054, USA

## Abstract

Isolated cranial nerve injury is a very rare complication of anesthesia. Specifically, hypoglossal nerve palsy affects mobility of the tongue and basic functions of swallowing and speech, and injury can be associated with placement and/or positioning of the endotracheal tube. Many etiologies are described that are unrelated to anesthesia such as tumors, stroke, trauma, or surgical dissection. Identification of hypoglossal neuropraxic-type injury from compression or stretching during anesthetic procedures can be difficult and tends to be a diagnosis of exclusion. Here, we present a case of a unilateral isolated hypoglossal nerve palsy following prolonged intubation in a surgery that involved large fluid shifts resulting in tongue swelling, in which establishment of the airway was initially difficult requiring two attempts. We suggest it is equally as possible that stretch injury occurred during airway instrumentation versus prolonged compression of the nerve between the endotracheal tube and the hyoid bone, possibly relating to a swollen tongue. We outline some treatments that have been used in previous reports and analyze their relation to improvements in symptoms. We conclude that instrumentation of the airway and prolonged intubation are both potential risk factors for hypoglossal nerve palsy, and identification of these risk factors can improve patient care by prompting patient discussions, guiding intraoperative management, and initiating earlier therapies.

## 1. Introduction

Undergoing a general anesthetic procedure involves the risk for many potential complications that can adversely affect a patient's perioperative experience. There is a spectrum of complications related to laryngeal manipulation and prolonged orotracheal intubation that include everything from minor cuts of the lip or oropharyngeal mucosa, dental injury, and even injury of nerves supplying structures of the oropharynx [1, 2].

The most common neurological injuries associated with laryngeal manipulation and intubation are injury of the oropharyngeal nerves including palsies of the recurrent laryngeal nerve, the hypoglossal nerve, and the external branch of the laryngeal nerve, otherwise known as the lingual nerve [3–7]. Three main physiologic functions of the muscles supplied by the hypoglossal nerve are swallowing, speech, and glottic control preventing aspiration [8]. Injuries to the hypoglossal nerve affect lingual muscles and, therefore, the ability to fully control mobility of the tongue, resulting in symptoms including dysarthria, dysphagia, and unilateral deviation of the tongue to the affected side with unilateral injury and inability to protrude the tongue with bilateral injury. Although these injuries have been reported in the literature, isolated unilateral iatrogenic hypoglossal nerve palsy is exceedingly rare and appears to be a diagnosis of exclusion after ruling out tumor, trauma, cerebral infarction, multiple sclerosis, and other neuropathies [5, 9, 10]. Neuropraxia can result from placement or positioning of the endotracheal tube, and even removal of the tube has been described [7]. Additionally, isolated injury to cranial nerves have been described by use of supraglottic airway devices [1, 9, 11] and even position of the chin-strap during shoulder surgery operations in the sitting position [6, 8, 12].

Hypoglossal nerve neuropraxia has been reported to resolve after a period of a few weeks [4], to as long as 4–6 months [3], and lingual nerve injuries resolved in an average of one month [2]. This duration of recovery may depend on early identification and possible intervention. Although minimal therapies have proven to be particularly useful, early treatment with dexamethasone has been described as aiding in the functional recovery from hypoglossal and lingual nerve injury resulting from intubation by decreasing neuroinflammation [10, 13]. Additional therapies including electrical stimulation, vitamin B12, and ear-nose- and throat-guided therapy have also been used to attempt to accelerate spontaneous recovery [10, 14]. However, none of these treatments have controlled studies demonstrating their benefit.

## 2. Case Report

A 59-year-old male presented for pancreaticoduodenectomy for T3N1 Pancreatic Adenocarcinoma. He was induced with 200 mg propofol, 70 mg lidocaine, 100 mcg fentanyl, and 60 mg rocuronium. Induction was completed successfully without any major hemodynamic instability facilitating endotracheal intubation. Following induction, the patient was easily masked without the need for an oropharyngeal airway. Upon initial attempt at intubation, a Macintosh size 4 laryngoscope blade was inserted successfully without obvious damage to oropharyngeal structures. Obtaining the view of the vocal cords was difficult as it was anteriorly displaced, and there was limited mobility of the larynx. Additional anterocaudal force was applied to the laryngoscope to attempt to improve view and laryngeal manipulation was required for a final best view of glottis structures to achieve a Cormack/Lehane score of grade 2B. At this time, the decision was made to abort direct laryngoscopy and proceed with video laryngoscopy for improved view.

The video laryngoscope size 4 blade was inserted without dental or oropharyngeal trauma and laryngeal manipulation was required for a final best view of glottis structures to achieve a Cormack/Lehane score of grade 2A. A 7.5 mm internal diameter, and a cuffed endotracheal tube was inserted without any mucosal trauma or bleeding and was placed posterolateral to the tongue and secured with tape at 22 cm at the teeth.

The intraoperative course was complicated by prolonged duration and excessive blood loss requiring large volume resuscitation to maintain hemodynamic stability. The surgery duration was 10 hours 55 minutes with an estimated blood loss of 1500 cc requiring a transfusion of 3 units of packed red blood cells, in addition to infusion of 8000 cc of crystalloid and 1000 cc of 5% albumin. There were no adjustments made to the positioning of the endotracheal tube of cuff pressure during the procedure. The patient was hemodynamically stable at the end of the surgery and was successfully extubated and taken to the general postanesthesia care unit where he was awake, responsive, and reported that his pain was adequately controlled. Upon waking up the following day, the patient first noticed slurred speech accompanying a swollen tongue and when examined by the bedside nurse was noted to have dysarthria and deviation of the tongue to the left. As the day progressed, the patient did notice improvement in his symptoms. Neurology was consulted and performed a complete neurological exam which was significant only for the findings described above. There was no word-finding difficulty, aphasia, weakness, or other cranial nerve or focal neurological deficits. The patient was alert and oriented to person, place, time, and situation. A MRI of the brain without contrast was performed to investigate possible cerebral infarction, which was ultimately negative. The neurology team concluded the deficit was likely hypoglossal nerve palsy related to prolonged orotracheal intubation.

Follow-up with the patient on postoperative day 2 showed improvements of approximately 75% in swelling, dysarthria, and deviation, although the symptoms were still present. Postoperative day 6 and day 17 showed similar symptoms as postop day 2, with the patient noting no additional improvements during these intervals. Working with speech pathology mildly improved speech; however, tongue deviation and pronunciation of specific letter sounds still proved difficult. Approximately 2 months after the procedure, the patient's symptoms completely resolved spontaneously.

## 3. Discussion

Hypoglossal nerve palsies, both isolated and in conjunction with other nerves, have been linked to numerous anesthesia-related iatrogenic etiologies including laryngeal manipulation during intubation or with ENT suspension cases, prolonged placement of the endotracheal tube [3, 5, 10], use of laryngeal mask airways [1, 9, 11], bronchoscopy [15], and shoulder surgery with utilization of chin straps [6, 8].

Considering the pathway of the hypoglossal nerve can assist in identifying mechanisms of isolated unilateral injury. As the nerve exits the medulla segment of the brainstem and courses through the hypoglossal canal, it passes between the major vessels in the neck and continues near the greater cornu of the hyoid bone. It then enters the mouth along the posterior margin of the mylohyoid muscle, which originates along the mandible and attaches to the hyoid bone [16]. The structures along this course leave the nerve vulnerable to injury from traumatic, forceful laryngeal manipulation, prolonged intubation, or extreme flexion and extension positioning of the neck. Previous reports have suspected both the laryngoscope blade and the cuff of the endotracheal tube stretching or compressing the nerve against the hyoid bone [3, 4, 7]. This abnormal force either via stretching or compressing the nerve between surrounding structures along its course is likely the underlying mechanism of injury.

In this case, it is unclear whether the injury occurred as a result of direct laryngoscopy with the additional force applied to the blade combined with external laryngeal manipulation during the initial attempt or due to the prolonged surgical procedure that involved major fluid shifts resulting in tongue swelling and compression of the nerve between the endotracheal tube and the hyoid bone. Despite the cause, this case demonstrates two mechanisms in which the injury could have occurred.

## 4. Conclusions

Isolated unilateral hypoglossal nerve palsy of iatrogenic etiology related to anesthetic management is a condition rarely reported in the literature, although the cases that have been reported have helped guide our understanding of the potential risk factors such as laryngeal manipulation and positioning of the endotracheal tube and patient [4, 6, 8, 12]. Identifying and reporting additional cases will help increase awareness of these risk factors and allow providers to be prepared and, perhaps, alter their plan for intubation and positioning. Additionally, it will assist with knowing when it is appropriate to have pre-emptive conversations with patients about what to expect in the event an injury should occur.

Knowledge of the most common risk factors can also guide management, such as checking intraoperative cuff pressures and performing postoperative exams seeking deficits in patients who have had traumatic airway manipulation or prolonged intubation without intermittent adjustment of the endotracheal tube during the operation. Establishing an injury has occurred early is important in order to rapidly initiate therapy such as dexamethasone or physiotherapy, which increases the likelihood of a more rapid and complete recovery [10, 13, 14]. Patients are often anxious and confused, often mistaking symptoms for a stroke, which is usually the leading differential diagnosis during initial workup [10]. As a diagnosis of exclusion, counseling patients on what to expect with regards to their treatment and rehabilitation and the likelihood of complete recovery is exceptionally important as they will have obvious symptoms without a clear diagnosis or demonstration of pathology on imaging.
